# Characterization of the complete mitochondrial genome of *Sinocyclocheilus lingyunensis* (Cypriniformes: Cyprinidae)

**DOI:** 10.1080/23802359.2021.1914222

**Published:** 2021-05-27

**Authors:** Peng Li, Jian Yang

**Affiliations:** Key Laboratory of Environment Change and Resources Use in Beibu Gulf, Nanning Normal University, Nanning, China

**Keywords:** Mitochondrial genome, *Sinocyclocheilus lingyunensis*, phylogeny

## Abstract

*Sinocyclocheilus lingyunensis* is endemic to Youjiang River in Guangxi, Southwestern China. In this study, the complete mitochondrial genome of *S. lingyunensis* was sequenced. It was determined to be 16,572 bases. The overall base composition was 31.9% A, 25.3% T, 26.9% C, 15.9% G with 42.8% GC content. The nucleotide sequence data of 12 heavy-strand protein-coding genes of *S. lingyunensis* and other 16 *Sinocyclocheilus* species were used for phylogenetic analyses. Trees constructed using Bayesian inference showed strong support for a topology indicating *Sinocyclocheilus* species as a monophyletic group.

*Sinocyclocheilus lingyunensis* Li (2000), an endemic cyprinid fish in China, was first found in the cave Shadong in Lingyun County and distributed in underground streams of the Youjiang River systems in Guangxi, China (Li et al. 2000; Lan et al. 2013). Body elongated, laterally compressed. Head small; snout blunt. Two long pairs of barbels. Body scales medium size, round, with 71–78 lateral line scales. Dorsal fin with seven-branched rays. Body cesious, some individuals light red. *Sinocyclocheilus lingyunensis* was similar to *S. jj,* but they can be distinguished by several morphological characters. For example, they had lateral line scales 71–78 and 48–50, respectively. *S lingyunensis* had harden and serrated of last unbranched dorsal ray, compare with smooth, slender and soft dorsal ray of *S. jj* (Zhang and Dai 1992; Li et al. 2000). In this study, the complete mitochondrial genome of *S. lingyunensis* was generated, then phylogenetic analysis of genus *Sinocyclocheilus* was performed using protein-coding genes in the mitochondrial genome. The sequencing of this mitogenome could facilitate future studies into the genetics and evolutionary history of this species.

The specimen of *S. lingyunensis* was collected from cave Shadong (belong to Youjiang River) in Lingyun County of Guangxi, China (24°20'N, 106°32'E). The voucher specimen was preserved in 95% ethanol and deposited in the Zoological Specimen Museum of Nanning Normal University (Jian Yang, yj1981yj@163.com), with an accession number NNNU20200901. Total genomic DNA was extracted using standard phenol/chloroform methods (Sambrook et al. 1989). The DNA library was constructed and sequenced using Illumina Hiseq 4000 platform (Illumina, San Diego, CA). Total of 16,512,684 reads (average trimmed length, 303.1 bp) were obtained after removing low DNA quality regions (below Q5) using fastp (Chen et al. 2018). The clean reads were assembled with SOAPdenovo v2.04 (Luo et al. 2012) and MITObim v1.6 (Christoph et al. 2013). The assembled genome was annotated using GeSeq (Tillich et al. 2017) and manually corrected, and then submitted into the GenBank database with accession number MW411665. Raw sequencing reads were deposited in NCBI SRA database with accession numbers PRJNA687907.

The complete mitochondrial genome of *S. lingyunensis* was 16,572 bp in length, which consisted of 13 protein-coding genes, 22 tRNA genes, and two rRNA genes, and a D-loop locus (control region). Its organization and gene order were similar to those of other teleosts mitochondrial genomes. The overall base composition was 31.9% A, 25.3% T, 26.9% C, 15.9% G with 42.8% GC content. The ND6 and 8 of the 22 tRNAs genes (tRNA^Gln^, tRNA^Ala^, tRNA^Asn^, tRNA^Cys^, tRNA^Tyr^, tRNA^Ser^(UCN), tRNA^Glu^, tRNA^Pro^) were encoded on the light strand, and the others were encoded on the heavy strand. COI gene started with GTG codon, while the other 12 protein-coding genes started with ATG. Seven of the protein-coding genes ended with complete stop codon (TAA or TAG), while 6 genes (ND2, COX2, COX3, ND3, ND4, and CYTB) used incomplete stop codon (T—).

To examine phylogenetic relationships in the genus *Sinocyclocheilu*s, phylogenetic trees were derived from the concatenated sequence of 12 protein-coding genes from 17 *Sinocyclocheilus* species and two outgroup species (*Cyprinus carpio carpio* and *Barbus barbu*) by employing Bayesian inference. Partitioned Bayesian phylogenetic analyses were conducted using MrBayes 3.1.2 (Ronquist and Huelsenbeck 2003). The best-fitting model (GTR + I + G) of sequence evolution for Bayesian analyses was obtained by Modeltest 3.7 (Posada and Crandall 1998) under the Akaike Information Criterion.

The phylogenetic result was similar to prior evolutionary relationships derived from molecular data (Li et al. 2017, 2018). It showed that *S. lingyunensis* was closely related to *S. ronganensis* and S*. yishanensis.* All *Sinocyclocheilus* species clustered as one monophyletic group and grouped into two major clades with strong support (Figure 1). *Sinocyclocheilu*s *jii* was the sister taxon to a clade of all other *Sinocyclocheilus* species examined in the phylogenetic reconstruction.

**Figure 1.**
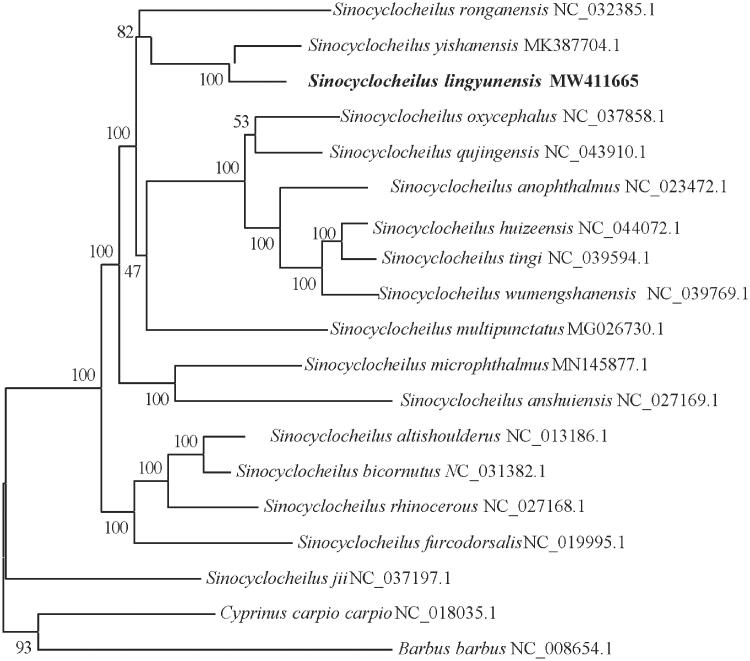
Bayesian 50% majority-rule consensus phylogenetic tree of *S. lingyunensis. Cyprinus carpio carpio* and *Barbus barbus* were used as outgroups. Numbers on the internode branches are Bayesian posterior probability.

## Data Availability

The data that support the findings of this study are openly available in GenBank of NCBI at https://www.ncbi.nlm.nih.gov, reference number MW411665.
